# Mutagenesis Study of the Cytochrome *c* Subunit Responsible for the Direct Electron Transfer-Type Catalytic Activity of FAD-Dependent Glucose Dehydrogenase

**DOI:** 10.3390/ijms19040931

**Published:** 2018-03-21

**Authors:** Yuki Yamashita, Nanoha Suzuki, Nana Hirose, Katsuhiro Kojima, Wakako Tsugawa, Koji Sode

**Affiliations:** 1 Department of Biotechnology and Life Science, Graduate School of Engineering, Tokyo University of Agriculture & Technology, Koganei, Tokyo 184-8588, Japan; 2 Ultizyme International Ltd., Meguro, Tokyo 152-0013, Japan; kojima@ultizyme.jp; 3 Joint Department of Biomedical Engineering, The University of North Carolina at Chapel Hill and North Carolina State University, Chapel Hill, NC 27599, USA

**Keywords:** glucose dehydrogenase, direct electron transfer, cytochrome *c*, glucose sensor, biomedical engineering

## Abstract

The FAD-dependent glucose dehydrogenase from *Burkholderia cepacia* (FADGDH) is a hetero-oligomeric enzyme that is capable of direct electron transfer (DET) with an electrode. The cytochrome *c* (cyt *c*) subunit, which possesses three hemes (heme 1, heme 2, and heme 3, from the N-terminal sequence), is known to enable DET; however, details of the electron transfer pathway remain unknown. A mutagenesis investigation of the heme axial ligands was carried out to elucidate the electron transfer pathway to the electron mediators and/or the electrode. The sixth axial ligand for each of the three heme irons, Met109, Met263, and Met386 were substituted with His. The catalytic activities of the wild-type (WT) and mutant enzymes were compared by investigating their dye-mediated dehydrogenase activities and their DET abilities toward the electrode. The results suggested that (1) heme 1 with Met109 as an axial ligand is mainly responsible for the electron transfer with electron acceptors in the solution, but not for the DET with the electrode; (2) heme 2 with Met263 is responsible for the DET-type reaction with the electrode; and (3) heme 3 with Met386 seemed to be the electron acceptor from the catalytic subunit. From these results, two electron transfer pathways were proposed depending on the electron acceptors. Electrons are transferred from the catalytic subunit to heme 3, then to heme 2, to heme 1 and, finally, to electron acceptors in solution. However, if the enzyme complex is immobilized on the electrode and is used as electron acceptors, electrons are passed to the electrode from heme 2.

## 1. Introduction

The monitoring of blood glucose is essential for diabetes therapy. Recently, more attention has been given to continuous glucose monitoring (CGM), in which the glucose concentration of interstitial fluid is measured continuously by a glucose sensor inserted subcutaneously or by non-invasive methods [1]. CGM together with the combination of sensor augmented insulin infusion technologies is currently the most advanced biomedical engineering in diabetes therapy. Recently, the U.S. Food and Drug Administration approved the first automated insulin delivery device for type 1 diabetes [2]. Conventional enzyme sensors require electron mediators to transfer electrons to the electrode. By contrast, DET-type enzyme sensors transfer electrons directly from the enzyme to the electrode. The DET principle enables the construction of lower potential sensors and avoids the use of toxic mediators in the system, thus making it attractive for implantable sensors. Glucose sensors employing enzymes that are capable of DET will be ideal for the application for CGM.

FAD-dependent glucose dehydrogenase isolated from *Burkholderia cepacia* (FADGDH) [3] is one of the limited glucose dehydrogenase enzymes capable of DET with an electrode. This enzyme is a hetero-oligomeric enzyme composed of three subunits: a catalytic subunit (α subunit) harboring an FAD cofactor, a small subunit (γ subunit) required for proper folding and secretion of the α subunit, and a cyt *c* subunit (β subunit), which harbors three hemes (heme 1, heme 2, and heme 3, from the N-terminal sequence). Cloning and functional expression in *Escherichia coli* (*E. coli*) was achieved [4,5], and protein engineering studies have been conducted to improve its substrate specificity, making this enzyme more suitable for glucose sensing [6,7]. Additionally, the development of several glucose sensing devices utilizing the DET capability of this enzyme have been reported [7,8,9,10]. Recently, the presence of a 3Fe–4S-type iron-sulfur cluster in the α subunit was elucidated [11]. The 3Fe–4S-type iron-sulfur cluster is the primary electron acceptor of FAD in the catalytic subunit, and it may play a role in the inter-molecular electron transfer from FAD to the multi-heme cyt *c* subunit.

To facilitate further engineering of FADGDH as a DET-type glucose sensor component, elucidation of the electron transfer pathway within this enzyme is necessary. Previous studies have revealed that when FADGDH is immobilized onto an electrode without electron mediators, the current response for glucose is drastically lowered in the absence of the β subunit [8], thus demonstrating that DET occurs via the β subunit. The spectrum unique to cyt *c* changed to its reduced form with the addition of glucose to the FADGDH solution [12]. From these results it is obvious that the electrons generated by the catalytic reaction of glucose oxidation are transferred from the α subunit to the β subunit. Combining these facts with the recent finding of the presence of the iron-sulfur cluster in the α subunit [11], it could be presumed that the electrons generated by the catalytic reactions are transferred from FAD through the iron-sulfur cluster to the β subunit and then to the electrode in the DET reaction. Although electrons are likely transferred to the electrode from one of the three covalently bound hemes of the β subunit, the detailed electron pathway within the β subunit remains unknown.

**Figure 1:**
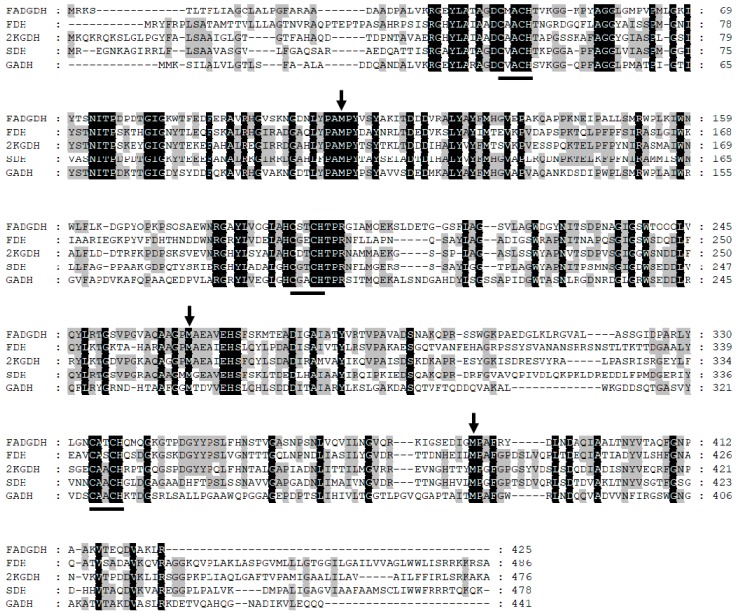
Primary structural alignment of the cyt *c* subunits of hetero-oligomeric dehydrogenases. The aligned sequences were cyt *c* subunits of; FADGDH: FADGDH from *Burkholderia cepacia* (GenBank accession no. AAQ06608), FDH: fructose dehydrogenase from *Gluconobacter japonicas* (GenBank accession no. BAM93251), 2KGDH: 2-ketogluconate dehydrogenase from *Gluconobacter japonicas* (GenBank accession no. BAQ21464), SDH: sorbitol dehydrogenase from *Gluconobacter frateurii* (GenBank accession no. BAD60914), and GADH: gluconate dehydrogenase from *Pantoea cypripedii* (GenBank accession no. AAC45884). These sequences were aligned and analyzed using Clustal Omega software [13]. The conserved Cys-Xaa-Xaa-Cys-His motifs are indicated by a black bar. The target Met residues (Met109, Met263, and Met386) are indicated by black arrows.

Elucidation of the inter/intra-electron transfer pathway in the β subunit will reveal the entire pathway from glucose oxidation to the external electron acceptor. To achieve this goal, a mutagenesis study of the residues proximal to the hemes was conducted. By comparing and analyzing the primary structures of the β subunit and the cyt *c* subunits of the reported hetero-oligomeric flavocytochrome dehydrogenases using the Clustal Omega software (Available online: https://www.ebi.ac.uk/Tools/msa/clustalo/, accessed on 17 Feb 2018) [13], the sixth ligand for each of the three heme irons was predicted (Figure 1). Consequently, Met109, Met263, and Met386 were substituted to His. The catalytic activities of the WT and mutant enzymes were compared by investigating their dye-mediated dehydrogenase activities and their DET abilities toward the electrode. Based on the obtained results, two different electron transfer pathways were proposed depending on the electron acceptor.

## 2. Results

### 2.1. Evaluation of the Mutants by Enzyme Activity Assay in Solution

The Cys-Xaa-Xaa-Cys-His motif is a typical cyt *c* heme-binding motif. Bacterial cyt *c* is maturated by the cyt *c* maturation system in the periplasm, in which the heme is covalently bound to the polypeptide by thioether bonds of the two Cys residues in the binding motif. The heme iron is six-coordinate, in which four ligands are provided by the nitrogen atoms of the porphyrin ring and two residues from the polypeptide provide the axial coordinates. The fifth ligand is the His of the heme binding motif, and the sixth residue is provided from the polypeptide chain, which is often a Met. As a fully-conserved Met was identified downstream of each of the three heme binding motifs, we presumed that these residues are the sixth ligands of the hemes. The corresponding Met residues in the β subunits were Met109, Met263, and Met386 (Figure 1). In this paper, the corresponding heme for each Met ligand, Met109, Met263, and Met386, will be referred to as heme 1, heme 2, and heme 3, respectively. Changing the sixth ligand from Met to His or His to Met was reported to change the redox potential of the corresponding heme without the significant distortion of protein structures [14,15,16,17,18]. Introduction of His mutations to the predicted sixth ligands of the β subunit might, therefore, have an effect on the intra/inter-molecular electron transfer efficiency, consequently affecting the activity while minimizing the impact on the 3D structure of the entire protein.

The mutants were evaluated by spectroscopic measurement, the spectrum of each mutant showed a spectrum characteristic to cyt *c*. Spectrum change could be observed after addition of glucose, showing the spectrum characteristic to the reduced form of cyt *c* (Figure S1). Therefore, the electron transfer to the β subunit was confirmed with all mutants.

**Figure 2:**
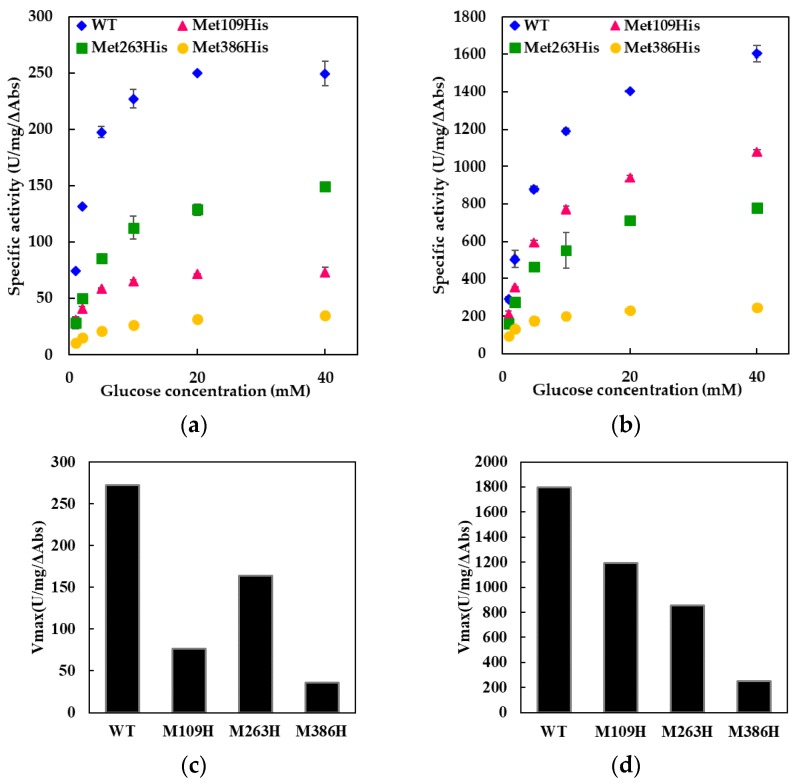
The dye-mediated dehydrogenase activities of WT and mutant FADGDHs. The activities were measured using either (**a**) 2-hexaammineruthenium(III) chloride (Ru-MTT) or (**b**) PMS (PMS-DCPIP) as an electron acceptor. Figure 2c,d are the comparison of Vmax values for WT and mutant FADGDHs using either (**c**) 2-hexaammineruthenium(III) chloride (Ru-MTT) or (**d**) PMS (PMS-DCPIP) as an electron acceptor.

To evaluate the enzymatic activities of the WT and constructed mutants, activity assays using two different electron acceptors, 2-hexaammineruthenium(III) chloride (Figure 2a) and PMS (Figure 2b), were performed. All mutants showed dye-mediated dehydrogenase activity dependent on the glucose concentration in both assays, but each mutant showed different dye-mediated dehydrogenase activity compared with the WT.

Decreases in the activities of all three mutants were observed for the assay using 2-hexaammineruthenium(III) chloride (Vmax: 273 U/mg/ΔAbs, 76 U/mg/ΔAbs, 164 U/mg/ΔAbs, and 36 U/mg/ΔAbs for WT, Met109His, Met263His, and Met386His). The mutation of each sixth ligand of the three hemes affected the electron transfer to 2-hexaammineruthenium(III) chloride in the β subunit, indicating the involvement of all three hemes in the electron transfer pathway. Significant decreases in activity were observed in Met109His and Met386His (Figure 2c). The impact of the mutation Met263His on activity was small compared to other mutants (60% of WT enzyme).

Decrease in the activities of all three mutants were also observed for the assay using PMS as electron acceptor (Vmax: 1790 U/mg/ΔAbs, 1190 U/mg/ΔAbs, 856 U/mg/ΔAbs, and 251 U/mg/ΔAbs for WT, Met109His, Met263His, and Met386His). Thus, the involvement of all three hemes in the electron transfer pathway was indicated also for this assay. Using PMS as the electron acceptor, Met386His showed a significant decrease in the activity, similar to those observed using 2-hexaammineruthenium(III) chloride, with an activity less than 14% of WT enzyme (Figure 2d). Met263His also showed a decrease in activity, but the impact was similar to that observed for the assay using 2-hexaammineruthenium(III) chloride (Figure 2d). Interestingly, Met109His showed different response toward the two different electron acceptors, the activity toward PMS was approximately 66% of that of the WT enzyme (Figure 2d) unlike the activity toward 2-hexaammineruthenium(III) chloride, which was 28% of that of the WT enzyme (Figure 2c).

These results indicated that the Met109His mutation resulted in a change in the mediator dependent dye-mediated dehydrogenase activity, but the Met263His mutation did not result in a significant change in dye-mediated dehydrogenase activity. However, Met386His resulted in a drastic decrease in the dye-mediated dehydrogenase activity regardless of the electron acceptors.

### 2.2. Evaluation of the Electron Transfer Efficiency by DET with an Electrode

**Figure 3:**
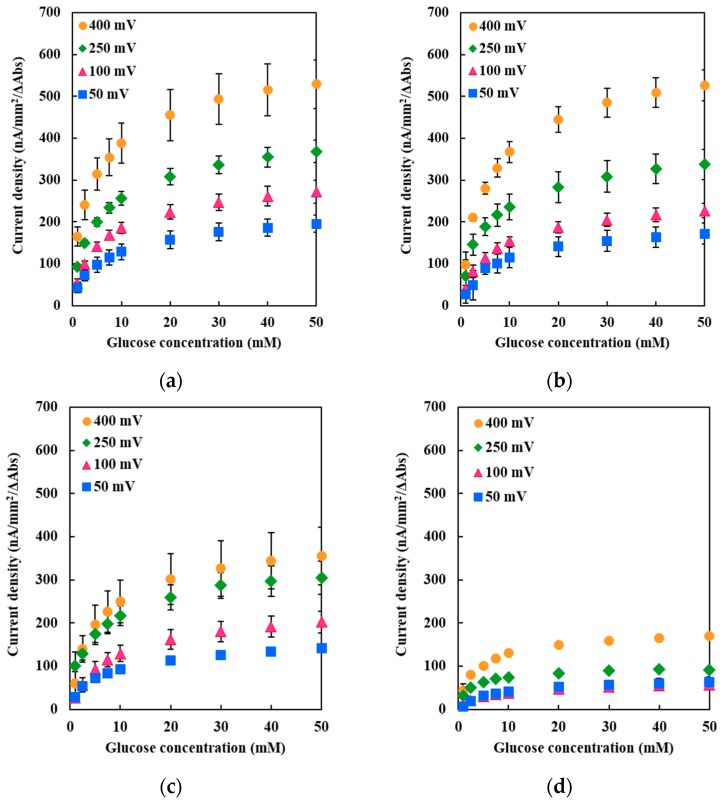
DET-type catalytic current measurement of the (**a**) WT; or mutant FADGDH (**b**) Met109His; (**c**) Met263His; and (**d**) Met386His-immobilized gold electrode. Buffer: 100 mM PPB (pH 7.0), 37 °C. Reference electrode: Ag/AgCl; counter electrode: Pt wire. Applied potential: +400, +250, +100, and +50 mV vs. Ag/AgCl.

To verify the effects of the mutations in DET-type electrocatalysis, the DET ability was evaluated by immobilizing each mutant on a glassy carbon electrode. The current densities under applied potentials of +50, +100, +250, and +400 mV vs. Ag/AgCl in the presence of various glucose concentrations were measured. A glucose concentration-dependent increase in the current density could be observed in all three mutants under all applied potentials (Figure 3b–d), indicating the occurrence of DET in all mutant-modified electrodes.

**Figure 4:**
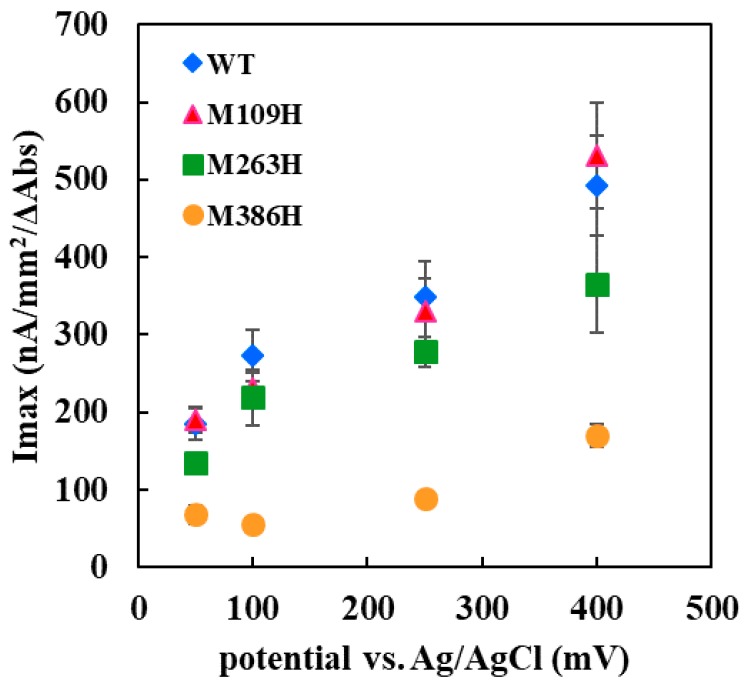
Imax value dependency on the applied potential vs. Ag/AgCl for the electrode immobilized with WT, Met109His, Met263His, or Met386His enzyme.

The Met109His-immobilized electrode showed a slightly lower DET response compared with the WT enzyme-immobilized electrode at all the applied potentials investigated in this study. Figure 4 shows the dependency of the Imax values on the applied potentials. The Imax value for each enzyme corresponds to the Vmax value calculated from the glucose concentration-dependent current density shown in Figure 3. The Imax values, as well as its dependency on the applied potentials of Met109His, were almost identical to those of the WT enzyme, indicating that the Met109His mutation did not affect the DET ability toward the electrode with this enzyme.

Interestingly, the Met263His-immobilized electrode showed a lower DET response compared with the WT enzyme-immobilized electrode, different from those of Met109His (Figure 3c). The Imax value dependency on the applied potential for Met263His was lower than that of WT and Met109His (Figure 4). Therefore, the Met263His mutation affected the DET ability, especially the applied potential dependency.

The Met386His-immobilized electrode showed a much lower DET response compared with the WT enzyme-immobilized electrode, independent of the applied potential (Figure 3d). The current density was approximately 15–33% of that of the WT enzyme-immobilized electrode, similar to the results of the dye-mediated dehydrogenase activity investigation of Met386His. Therefore, the Met386His mutation negatively affected not only the dye-mediated dehydrogenase activity, but also the DET ability.

## 3. Discussion

In this study, we aimed to elucidate the inter/intra-electron transfer pathway in the β subunit of FADGDH. To the best of our knowledge, no 3D structure of FAD dependent dehydrogenase composed of cyt *c* subunit was elucidated. In addition, no homologous structure to the β subunit of FADGDH was reported. However, the analysis of FADGDH has revealed that the dissociation of β subunit occurs after heat treatment resulting in the decrease of enzymatic activity showing that the β subunit plays an important role in the electron transfer between α subunit and the artificial electron mediator [12]. Cross-linking of the subunits of FADGDH resulted in high thermal stability without loss of catalytic activity supporting this hypothesis [19]. Therefore, the formation of quaternary structure of FADGDH is inevitable to show its characteristic electron transfer via cyt *c* subunit.

Throughout our experiments using FADGDH with electron transfer subunit, no precipitation was observed. The mutated electron transfer subunits were properly folded, thereby forming active quaternary structure with catalytic subunit. This was confirmed by the observation of the electron transfer reaction by the oxidation of glucose at the catalytic subunit resulting in reduction of electron acceptor, especially 2-hexaamineruthenium(III) chloride. In the activity assay using 2-hexaamineruthenium(III) chloride as an electron acceptor, the electrons can only be transferred from the β subunit, but not from catalytic subunit alone (Table S1; the comparison of dye-mediated dehydrogenase activities of catalytic subunit (γα), catalytic subunit with wild-type electron transfer subunit (β), and of catalytic subunit with mutated electron transfer subunits (β)). With the catalytic subunit alone, the dehydrogenase activities using 2-hexaamineruthenium(III) chloride was almost negligible compared with those using PMS-DCPIP. In contrary, the dehydrogenase activity of the catalytic subunit with the electron transfer subunit using 2-hexaamineruthenium(III) chloride was about 18% for wild type, 7.7% for Met109His mutant, 18% for Met263His mutant, and 14% for Met386His mutant compared with the assay using PMS-DCPIP. Thus, it is obvious that 2-hexaamineruthenium(III) chloride-dependent dye mediated dehydrogenase activity can only be seen when the catalytic subunit is forming a complex with the β subunit. We believe that no enzyme quaternary structure will be formed if the electron transfer subunit is not correctly folded. Therefore, these results simultaneously suggested that all mutant electron transfer subunits were folded properly to form quaternary structure, and thereby showed 2-hexaamineruthenium(III) chloride dependent dehydrogenase activity.

The Met109His mutation on the sixth ligand of heme 1 of FADGDH resulted in a change in the mediator dependent dye-mediated dehydrogenase, but did not affect the DET ability toward the electrode of this enzyme. The Met263His mutation on the sixth ligand of heme 2 of FADGDH did not result in a significant change in the mediator dependent dye-mediated dehydrogenase activity, whereas the Met263His mutation affected the DET ability, especially the applied potential dependency. Considering that the reaction conditions of enzyme with electrodes themselves are artificial, and would vary depending on the way of immobilization and the electrode materials, this enzyme complex originally may transfer electron via heme 1, and the role of heme 2 to transfer electron to electrode is observed only at the artificial conditions

The Met386His mutation on the sixth ligand of heme 3 of FADGDH resulted not only in a drastic decrease in the dye-mediated dehydrogenase activity regardless of the electron acceptors, but also the DET ability. The β subunit is essential to show 2-hexaammineruthenium(III) chloride-mediated dehydrogenase activity. Since this activity was obviously high compared with the FADGDH without β subunit (Table S1), it is evident that the Met386His mutant accepts electron from catalytic subunit by retaining its quaternary structure, and the electrons are transferred to external electron acceptor via β subunit.

The dye-mediated dehydrogenase activity was affected by the mutation in all three constructed mutants, it could be presumed that all hemes in the β subunit are involved in the electron transfer in the β subunit. Since the catalytic reaction occurs at the α subunit, the electrons are passed on to the β subunit and then to the external electron acceptors. The information allowed us to give an assumption that each heme of the FADGDH β subunit should have different roles; one responsible for the electron acceptor from the catalytic subunit, another one responsible for internal electron transfer and the last one responsible for the electron transfer to the external electron acceptor.

Only the mutation in the sixth ligand of heme 1 affected the response toward the two different electron acceptors used for the dye-mediated dehydrogenase assays. As the mutation in heme responsible for the electron transfer to the external electron acceptors might cause such an alteration, heme 1 was presumed to be mainly responsible to donate electron to the external electron acceptors in solution. Surprisingly, the DET ability observed for the electrode immobilized with the Met109His mutant was not affected by the mutation. Thus, the assumption that heme 1 is not involved in the DET could be made. If the mutation at the electron accepting heme resulted in a negative impact on the electron transfer from catalytic subunit, the effect should appear both in dye-mediated dehydrogenase activity and DET ability. The effect of the mutation in the sixth ligand of heme 3 resulted in a drastic decrease in the dye-mediated dehydrogenase activity and also the DET ability. Since Met109His mutation had a negative effect on dye-mediated dehydrogenase activity, but did not affect the DET ability, heme 1 is not the electron accepting heme. The Met263His mutation also had negative impact on both the dye-mediated dehydrogenase activity and the DET ability but the Met386His mutation had more drastic effect. Therefore, it was presumed that heme 3 may be responsible for accepting electrons from the α subunit. Since the presence of 3Fe–4S-type iron-sulfur cluster in the α subunit is reported in previous studies [11], the electrons may be transferred via the iron-sulfur cluster. This mutation may have caused a shift in the redox potential of heme 3, thus reduced the internal electron transfer efficiency. Although the mutation in the sixth ligand of heme 2 showed a slight decrease in the dye-mediated dehydrogenase activity, the impact was the same toward both electron acceptors investigated in this study, unlike for Met109His. Therefore, heme 2 does not seem to be the main heme responsible for external electron transfer in solution but is only responsible for internal electron transfer. However, Met263His affected the DET ability, especially the applied potential dependency. From these observations, heme 2 could be presumed to be the heme responsible for DET to the electrode.

**Figure 5:**
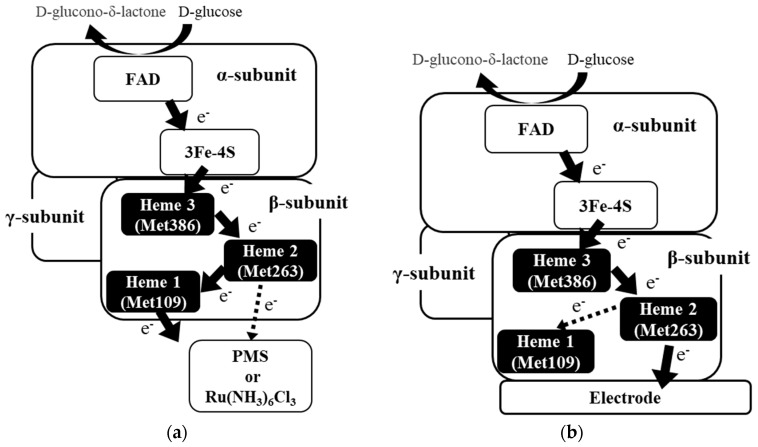
Schematic of the proposed electron transfer pathway of FADGDH. (**a**) Electron acceptors in a dye-mediated dehydrogenase activity assay in a solution; and (**b**) DET-type electron transfer to the electrode.

Considering these observations, the following two schemes for the electron transfer pathway are proposed, which are dependent on the availability of electron acceptors, soluble electron acceptors (Figure 5a) or an electrode for direct electron transfer (Figure 5b). First pathway is the electron transfer to electron acceptors dissolved in solution. In this pathway, the electrons are first passed from the α subunit via the iron sulfur cluster to heme 3, then to heme 2, and finally to heme 1. Although we cannot rule out the possibility that heme 2 may transfer electron directly to the external electron acceptor, heme 1 is the main electron-donating heme for the external electron acceptor in this pathway. The second pathway is the electron transfer pathway for DET electrocatalysis. The electron from the α subunit is also transferred through the iron-sulfur cluster to heme 3 in this pathway and then to heme 2. Heme 1 may accept an electron from heme 2. but it is not involved in DET with the electrode, and heme 2 is the heme responsible for electron transfer to the electrode.

The electron transfer pathway for DET-type bioelectrocatalysis for the cyt *c* subunit of fructose dehydrogenase, which is also a hetero-oligomeric flavocytochrome dehydrogenase, has been reported recently [20,21,22]. Through these studies, the authors concluded that the heme 2 is responsible for DET, without the contribution of heme 1. These results are in agreement with the results obtained in this study for FADGDH. Although the study of fructose dehydrogenase did not refer to the role of heme 1, our results indicated that heme 1 in fructose dehydrogenase might be involved in the electron transfer pathway in solution.

The present findings will provide valuable insights into understanding the DET mechanism of FADGDH, which is necessary for the further improvement of the performance and its application toward CGM systems and other DET-based glucose monitoring.

## 4. Materials and Methods

### 4.1. Chemicals

Phenazine methosulfate (PMS), D-glucose, and 2,6-dichlorophenolindophenol (DCPIP) were purchased from the Kanto Chemical Co., Ltd. (Tokyo, Japan). Ketjenblack EC600JT was purchased from the Lion Specialty Chemicals Co., Ltd. (Tokyo, Japan). Glutaraldehyde solution (25%, *w*/*v*) was purchased from Wako Pure Chemical Industries, Ltd. (Osaka, Japan). Nafion perfluorinated resin solution, 3-(4,5-dimethyl-2-thiazolyl)-2,5-diphenyl-2*H*-tetrazolium bromide (MTT) and 2-hexaammineruthenium(III) chloride were purchased from Sigma-Aldrich Japan G. K. (Tokyo, Japan). All other chemicals were of reagent grade.

### 4.2. Site-Directed Mutagenesis

Site-directed mutagenesis was carried out by overlap PCR using pTrcγαATGβ [5], the expression vector for the FADGDH γαβ complex, as a template. The resulting mutated PCR fragments and pTrcγαATGβ were cut by the restriction enzymes *Eco*RI and *Not*I, followed by ligation. All mutations were confirmed by nucleotide sequencing.

### 4.3. Preparation of FADGDH WT, Met109His, Met263His and Met386His

Each of the WT and mutated pTrcγαATGβ expression vectors was co-transformed into *E. coli* BL21(DE3) with the pBBJMccm [5] vector, which encodes the *E. coli* ccmABCDEFGH genes. The co-transformed *E. coli* were grown aerobically at 37 °C for 30 h in ZYP-5052 auto-induction medium (0.5% glycerol, 0.05% glucose, 0.2% lactose, 50 mM (NH_4_)PO_4_, 50 mM Na_2_HPO_4_, and 1 mM MgSO_4_) [23] containing 100 μg/mL ampicillin and 50 μg/mL kanamycin. The harvested cells were washed with 0.85% (*w*/*v*) NaCl, resuspended in 10 mM potassium phosphate buffer (PPB) at a pH of 7.0 and lysed by French pressing. Lysates were centrifuged at 9000× *g* at 4 °C for 15 min, and the obtained supernatants were centrifuged again at 104,000× *g* at 4 °C for 1 h. The pellets were suspended in 1 mL of 10 mM PPB (pH 7.0) containing 1.5% (*w*/*v*) sodium cholate and 0.1 M KCl per 0.5 g of pellet and solubilized at 1600 rpm for 1 h at 4 °C. The solubilized samples were centrifuged at 104,000× *g* at 4 °C, and crude extracts were obtained by dialyzing against 10 mM PPB at a pH of 7.0.

FADGDH was purified by hydrophobic interaction chromatography. The crude extracts were applied onto a 5 mL Hitrap Octyl FF Column (GE Healthcare Japan Co., Tokyo, Japan) equilibrated with 10 mM PPB (pH 7.0) and eluted using six column volumes of 10 mM PPB (pH 7.0) containing 0.4% (*w*/*v*) sodium cholate, followed by three column volumes of 10 mM PPB (pH 7.0) containing 1.0% (*w*/*v*) sodium cholate. The active fractions with absorbance at 410 nm were pooled and dialyzed against 10 mM PPB (pH 7.0).

### 4.4. Enzyme Activity Assay

The specific activities of FADGDH (WT or mutants) toward various concentrations of glucose were evaluated using two different mediators as the primary external electron acceptors. The electron mediators used were PMS and 2-hexaammineruthenium(III) chloride.

The activity assay using PMS as the electron mediator was carried out using color to indicate the second electron acceptor, DCPIP, by monitoring the decrease in the absorbance of DCPIP at 600 nm as described previously [11], except the assay buffer contained 0.2% Triton X-100.

The activity assay using the 2-hexaammineruthenium(III) chloride as the electron mediator was carried out in 10 mM PPB (pH 7.0) containing 0.2% Triton X-100 at room temperature with the presence of 1 mM MTT as a color indicator of the second electron acceptor, 2% 2-hexaammineruthenium(III) chloride with various concentrations of glucose. The increase in the absorbance of reduced MTT at 565 nm was recorded.

In both assays one unit (U) of GDH activity was defined as the amount that oxidized 1 μmol of glucose per minute under the standard assay conditions using the millimolar absorption coefficient of 16.3 mM/cm for DCPIP and 20 mM/cm for MTT. In both assays, the protein concentration was measured using a Bio-Rad protein assay (Bio-Rad, Hercules, CA, USA) kit to calculate the specific activity (U/mg).

To better evaluate the effects of mutations on the function of the β subunit, the results from the activity assay was normalized via the absorbance at the soret band (410 nm) of each enzyme (WT and mutants). The specific activity is indicated as U/mg/ΔAbs.

### 4.5. Electrode Preparation

The purified FADGDH (WT or mutants) was diluted to 0.2 mg/mL by 100 mM PPB (pH 7.0). This solution, 5% (*w*/*v*) Nafion and Ketjenblack EC600JT dispersed in 0.8% Triton X-100 were mixed together in a ratio of 8:1:1, respectively. One microliter of this mixture was applied onto a glassy carbon electrode (7 mm^2^) five times to apply 5 µL in total. The electrode was air-dried at 4 °C degrees for 1 h and was then placed in a sealed container filled with vapor from 25% (*w*/*v*) glutaraldehyde for 90 min of cross-linking. The electrode was stored at 25 °C until use.

### 4.6. Electrochemical Measurements

Chronoamperometry was carried out with the constructed enzyme electrodes in a three-electrode system: FADGDH (WT or mutants), as the immobilized working electrode; Ag/AgCl (3 M NaCl), as the reference electrode (model RE-1, BAS, Tokyo, Japan); and Pt wire, as the counter electrode. All three electrodes were placed in a water jacket cell filled with 10 mL of reaction buffer and 100 mM PPB (pH 7.0), and they were kept at 37 °C. The reaction buffer was continuously stirred at 250 rpm during the measurement. Potentials of +50, +100, +250, and +400 mV vs. Ag/AgCl were applied, and the current responses with the presence of various glucose concentrations (1, 2.5, 5, 7.5, 10, 20, 30 and 40 mM) in the reaction buffer were measured. The glucose concentration was increased by adding the glucose solution to the reaction buffer. The current density was calculated from the area of the working electrode (7 mm^2^).

The results for electrochemical measurements were also normalized via the absorbance at the soret band of each enzyme as described previously in Section 4.4. The current density is indicated as nA/mm^2^/ΔAbs.

## Supplementary Materials

Supplementary materials can be found at http://www.mdpi.com/1422-0067/19/4/931/s1.

## Abbreviations

FAD	flavin adenine dinucleotide
FADGDH	FAD-dependent glucose dehydrogenase isolated from *Burkholderia cepacia*
DET	direct electron transfer
Cyt *c*	cytochrome *c*
DCPIP	2,6-dichlorophenolindophenol
PMS	phenazine methosulfate
MTT	3-(4,5-dimethyl-2-thiazolyl)-2,5-diphenyl-2*H*-tetrazolium bromide
