# Supplementary material for: Mutagenesis Study of the Cytochrome c Subunit Responsible for the Direct Electron Transfer-Type Catalytic Activity of FAD-Dependent Glucose Dehydrogenase
Source: Int J Mol Sci. 2018 Mar 21;19(4):931. doi: 10.3390/ijms19040931 (PMC5979317; doi:10.3390/ijms19040931)
Supplement: Supplementary file 1 [file ijms-19-00931-s001.pdf]

Supplementary Materials:

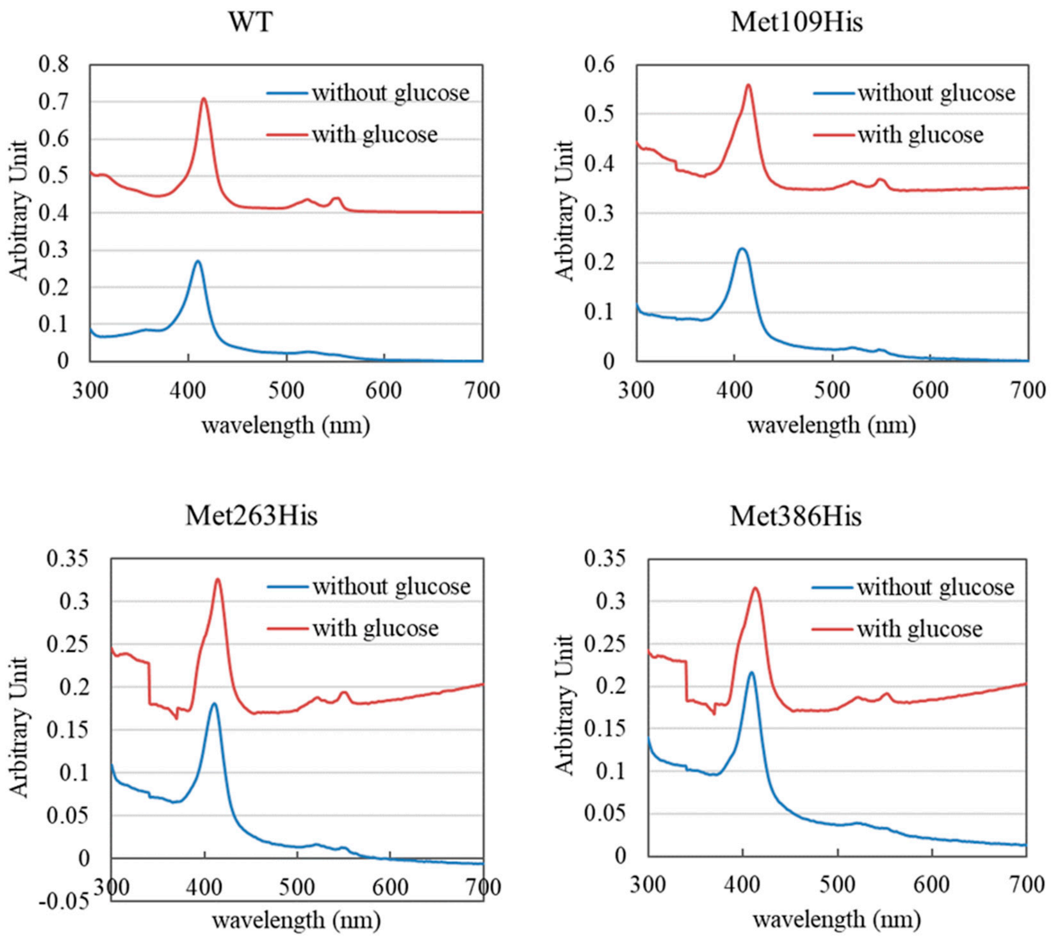

Supplemental Figure S1. Comparison of the absorbance spectrum before and after the addition of glucose. Blue line: without glucose; the absorbance spectrum (300–700 nm) was measured for WT and mutants (Met109His, Met263His, Met386His) before addition of glucose. Red line: with glucose; 100 mM glucose was added to each enzyme solution (WT and Met109His, Met263His, Met386His mutants) and incubated at room temperature for 30 minutes and the absorbance spectrum (300–700 nm) was measured. For better visualization, 0.4 was added to the original absorbance for with glucose data.

Supplemental Table S1. The comparison of dye-mediated dehydrogenase activities of the catalytic subunit ($\gamma\alpha$), catalytic subunit with wild-type electron transfer subunit (β), and of the catalytic subunit with mutated electron transfer subunits (β).

	Dye-mediated dehydrogenase activity		
	A: 2-hexaamineruthenium(III) chloride	B: PMS	A/B (%)
$\gamma\alpha$ complex※1	1.6×10^{-6} U/mg	190 U/mg	$1.6{\times}10^{-7}$%
WT $\gamma\alpha\beta$ complex	250 U/mg/ΔAbs	1400 U/mg/ΔAbs	18%
Met109His $\gamma\alpha\beta$ complex	72 U/mg/ΔAbs	940 U/mg/ΔAbs	7.7%
Met263His $\gamma\alpha\beta$ complex	130 U/mg/ΔAbs	710 U/mg/ΔAbs	18%
Met386His $\gamma\alpha\beta$ complex	32 U/mg/ΔAbs	230 U/mg/ΔAbs	14%

Activity assay was carried out in the presence of 20 mM glucose. Assay conditions are described in Section 4.4 except for 2-hexaamineruthenium(III)chloride was 80 mM for the $\gamma\alpha$ complex and assay was carried out in 10 mM PPB (pH6.0) for the $\gamma\alpha$ complex (millimolar absorption coefficient; DCPIP: 4.76 mM/cm, MTT: 20 mM/cm for MTT) ※1: FADGDH $\gamma\alpha$ complex was prepared as follows: The pTrc$\gamma\alpha$ expression vector was transformed into *E. coli* BL21. The transformed *E. coli* were grown aerobically at 20 °C for 30 h in ZYP-5052 auto-induction medium (0.5% glycerol, 0.05% glucose, 0.2% lactose, 50 mM $(NH_4)PO_4$, 50 mM Na_2HPO_4, and 1 mM $MgSO_4$) [23] containing 100 μg/ml ampicillin. The harvested cells were resuspended in 10 mM PPB (pH 6.0) and lysed by French press. The lysate was ultracentrifuged and the supernatant was collected. The collected supernatant was dialyzed against 10 mM PPB (pH 6.0) and equal amount of 10 mM PPB (pH 6.0) containing 2 M ammonium sulfate was added. The precipitate was excluded by centrifugation at 10,000× *g* for 10 minutes and the supernatant was collected. The collected supernatant was applied to Resource 15 PHE column (GE Healthcare Japan Co., Tokyo, Japan) equilibrated with 10 mM PPB (pH 6.0) containing 1 M ammonium sulfate. Elution was carried out using 10 mM PPB (pH 6.0) in linear gradient (0–100%) in 20 column volumes. The active fraction was pooled and concentrated by ultrafiltration after dialysis against 10 mM PPB (pH 6.0).
